# Web-Based System Navigation Database to Support Equitable Access to Assistive Technology: Usability Testing Study

**DOI:** 10.2196/36949

**Published:** 2022-11-03

**Authors:** Tamika Jarvis, Allison M L Mah, Rosalie H Wang, Michael G Wilson

**Affiliations:** 1 Department of Health Research Methods, Evidence, and Impact McMaster University Hamilton, ON Canada; 2 Department of Occupational Science & Occupational Therapy University of Toronto Toronto, ON Canada; 3 McMaster Health Forum McMaster University Hamilton, ON Canada; 4 Centre for Health Economics and Policy Analysis Hamilton, ON Canada

**Keywords:** assistive technology, program funding, usability testing, internet, web-based database, health services

## Abstract

**Background:**

Assistive technology (AT) can contribute to how individuals participate and engage in everyday activities, such as communication and mobility, and facilitates access to the services they require. Navigating Canada’s AT system has been described as fragmented and complex, presenting barriers for individuals who require AT, caregivers, and health service providers. AccessATCanada was developed as a centralized web-based resource to help support access to AT by providing information about the existing jurisdictional funding programs and services.

**Objective:**

This study aimed to evaluate the usability of AccessATCanada by gathering feedback about its features, functionality, and areas of strength and opportunity from potential end users.

**Methods:**

A usability testing study using a think-aloud approach and semistructured interviews was conducted to measure the effectiveness and efficiency of and user satisfaction with AccessATCanada and to identify issues with the interface during end-user interaction. A qualitative thematic analysis was used to generate insights into and core themes about user experiences. User feedback was used to inform subsequent updates of the database with the goal of enhancing website friendliness and functionality before its official launch.

**Results:**

A total of 10 participants (6 consumers, 1 caregiver, and 3 providers) participated in the usability testing study. The usability performance and scores tended to improve between the 2 testing cycles. Most participants were able to successfully complete all the tasks independently. The efficiency scores tended to improve as the users continued to engage with the interface. The website received an overall System Usability Score of 62.22, which was ranked as “OK/fair to good.” The users provided an overall positive evaluation of the beta version of the web-based resource tested over 2 cycles and helped to identify areas for improvement. They commented on the functionality and added value of the website, discovery of new programs and resources, and design aesthetics. Most usability issues were reported as minor challenges related to presentation, functionality, and language, and feedback was adopted into later iterations of the website.

**Conclusions:**

This study provides reflections on the value of usability testing and elements that are key to the creation of user-centered resources, such as the inclusion of participants with various abilities and considerations regarding website design and accessibility in an increasingly web-based world. AccessATCanada is now part of a growing global response to expand the reach of AT programs and services, improve the equity of access to AT, and reduce the complexity of navigating AT systems.

## Introduction

### Background

Assistive technology (AT) contributes to how an individual participates and engages in their everyday activities, such as communication and mobility, and facilitates access to the services that they require [1]. Access to AT, which promotes the inclusion, engagement, and participation of the world’s growing aging population and population of persons with disabilities, remains challenging. According to the World Health Organization, an estimated 90% of individuals who are in need of AT do not have access to it [2]. As noted in the “Global Report on Assistive Technology,” there are 3 phases in the pathway to access AT: seeking, obtaining, and realizing [3]. Seeking encompasses the first steps taken to enhance access to AT and necessitates that consumers, caregivers, and providers are informed about the available AT and can find and obtain related information. Successful access to information provides a foundation for individuals to continue accessing appropriate AT, regardless of whether the need is acute or long term.

More than 220 government and charitable organizational programs that provide funding and services for AT exist across Canada [1]. Access to AT programs has been described as fragmented and complex to navigate and uncoordinated between national, subnational, and local levels [4]. Adding to this complexity is the fact that programs are highly variable regarding the range of ATs that can be covered, eligibility criteria, and service-delivery mechanisms used [5]. Some Canadian jurisdictions, such as British Columbia, have introduced “one-stop” approaches or single-entry point systems, which are typically organizations that perform a range of activities, such as assessments, training, and AT, as well as manage access to funding sources [6]. However, these systems are far from being universal. Despite the many programs available for AT, poor “consistency in the quality and quantity of AT information,” the high cost of AT, and the lack of governmental funding support create difficulties with navigating programs and make the acquisition of AT challenging for those who need them. In addition, the lack of training presents significant barriers for health service providers in recommending appropriate AT [7].

The lack of a user-friendly system is a challenge that needs to be addressed in a way that is efficient, effective, and satisfactory to help reduce the impact of inequitable access to needed supports. These findings motivated the development of AccessATCanada, a web-based resource designed to be easily searchable by various users with different abilities. Program information for the database was initially gathered through a jurisdictional scan to identify the types of AT covered under jurisdictional programs and funding, eligibility criteria for AT programs, and currently available AT funding and services [1]. The database contents and website were updated before launch in January 2021. The website was created in compliance with Web Content Accessibility Guidelines (WCAG) 2.0 by a professional website development company.

**Figure 1:**
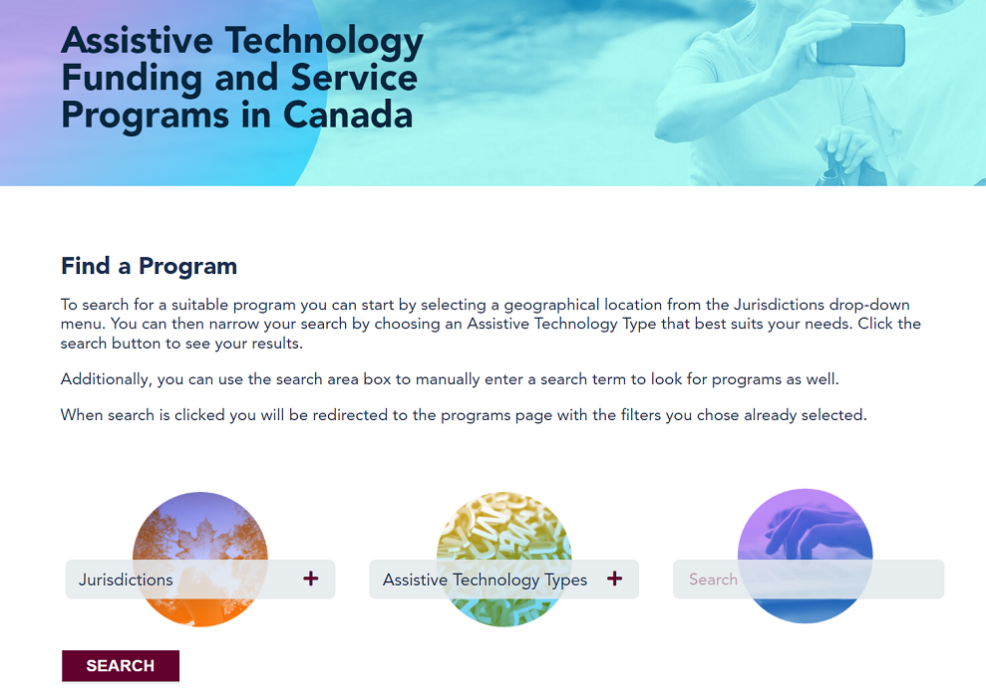
AccessATCanada Home page.

The Home page of the website allows users to search by keywords and has filters to search by jurisdiction, and AT types. It also features an interactive map of Canada, which allows users to search by province or territory (Figure 1). In addition, a feedback form is provided at the bottom of the page to allow users to report errors. The menu at the top of the Home page directs users to information about the website and collaborators (About Us page); a Programs page for users to find AT programs; a Resources page, which provides reports and publications related to AT in Canada; and a Contact Us page, where users are able to contact the project leads and provide feedback about the website.

### Objectives

The objective of this study was to examine the usability of the website and the interaction of a representative set of users with the website to further develop and refine a user-friendly resource that meets the needs of the end users. The process used to gather feedback, identify elements considered important to users, and refine the AccessATCanada website can help further enhance this resource and help guide those looking to create similar resources [8].

## Methods

This usability testing study used a think-aloud approach to gather feedback from prospective users related to effectiveness, user efficiency, and satisfaction with the website and to identify any potential issues with the interface [9].

### Recruitment

A purposive sample of participants from 3 groups of anticipated users were recruited: (1) consumers, defined as individuals who self-identified as living with at least one type of disability (eg, a physical, sensory, cognitive, or mental health limitation) and who may benefit from AT use; (2) caregivers, who identified as individuals who cared for someone who required or used AT; and (3) health care providers, community social services providers, and industry vendors, who identified as individuals who assist others in accessing AT. Although the aim was to recruit approximately 5 individuals from each user group, the authors found that there was a sufficient amount of information gathered to make immediate updates to the website with a smaller number of participants. The participants were required to have internet access via a desktop or laptop computer with screen sharing to capture immediate responses when using the website and for the researchers to assist with troubleshooting as needed. The participants were recruited through project partners who shared recruitment advertisements with those who used their services and their networks and informal networks of service providers.

### Ethics Approval

The study was approved by the Hamilton Integrated Research Ethics Board (REB-8325) and the University of Toronto Research Ethics Board (REB-38715). Informed consent was provided by all the participants.

### Data Collection

**Figure 2:**
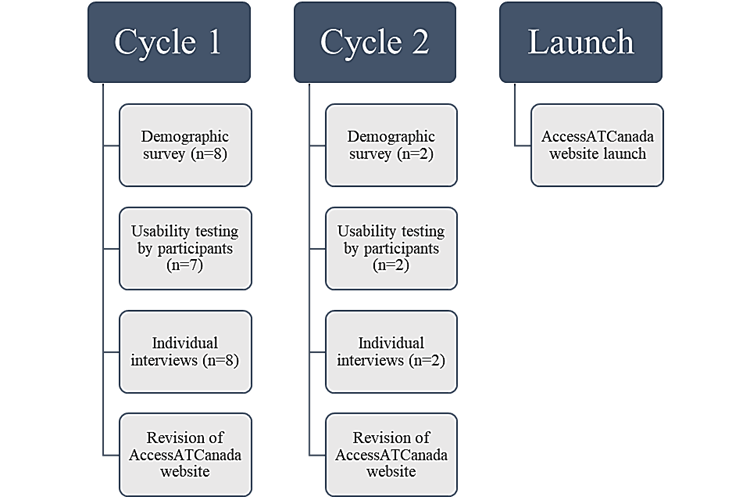
Diagram depicting the cycles of the usability testing procedure and website development.

Overall, 2 iterative cycles of task-based usability testing evaluations were conducted using an unreleased beta version of the AccessATCanada website. A total of 21 tasks were given to the participants, across 4 mock case scenarios (Multimedia Appendix 1). The usability testing sessions were facilitated by 2 members of the research team (AMLM and TJ). The usability testing sessions were conducted individually with each participant, lasting approximately 60 to 90 minutes in duration and audio-visually recorded through institutionally licensed WebEx (Cisco Systems, Inc) and Zoom (Zoom Video Communications) videoconferencing software. Figure 2 illustrates the cycles of usability testing and iterative refinement of the website. Usability testing included the completion of mock case scenarios, System Usability Scale (SUS), and a brief semistructured interview [10].

Before usability testing, the participants were asked to complete a brief demographic survey and to self-identify as a consumer, caregiver, or health care provider. Mock case scenarios were pilot tested with a member of the research team before data collection. They were written with the goal of obtaining information from diverse content searches within the website and required the participants to explore and use different website functions. The participants were asked to explore different provincial and territorial AT programs, navigate between government and charitable programs, find eligibility criteria for a funding service, and locate programs that funded specific AT needs. The scenarios and tasks were read aloud by the researcher in the same sequence for each participant. Notably, some tasks were performed out of order or were completed simultaneously by some participants unprompted; therefore, these tasks were skipped to avoid task repetition. The participants were prompted to think aloud and verbalize their actions and thoughts as they interacted with the system, which allowed for observation and real-time feedback [11]. To help identify areas for improvement, usability issues and errors (eg, issues related to functionality, presentation, and language and events that impacted the ability to use the website effectively and efficiently) were noted during testing sessions [12,13]. Approximately 60 minutes were allotted to completing the 4 mock scenarios; however, not all participants completed the tasks because of time constraints or technological challenges that arose during user testing sessions.

Following the completion of the mock scenarios, each participant was asked to complete the SUS. The SUS is a widely used and highly rated user-centered questionnaire that includes questions related to the learnability and complexity of and satisfaction with website use [10,14]. The SUS measures user comfort, satisfaction, and perception of usability of the website by verbally ranking their agreement with 10 statements on a 5-point Likert scale (Multimedia Appendix 2). After the completion of the SUS, a brief semistructured individual interview was conducted to elicit further clarification and elaboration of participants’ responses and experience while using the website, elaborate on their SUS ratings, and describe the website features that they liked the best and least. The interview guide can be found in Multimedia Appendix 3.

Participants’ observations and comments were compiled into interim summary reports between each usability testing cycle, which informed and optimized the next website iteration. A final report was then compiled based on the feedback provided by the second cycle of participants, which informed the recommended changes before the official launch of the website.

### Data Analysis

The assessment parameters and metrics of effectiveness, efficiency, and satisfaction were guided by the International Organization for Standardization 9241-11 [9]. All the tasks attempted were included in the data analysis.

Effectiveness was calculated and defined as the successful completion of tasks and scenarios [15]. The tasks that the participants were unable to complete or were completed with the assistance of the researcher were graded as “failure (not completed)” and given a score of 0, and the tasks that were completed with ease or with no assistance were graded as “success” and given a score of 1. The average effectiveness was calculated based on task failures or full successes among the attempted tasks. Tasks that were not attempted by the user were excluded from the data analysis. Usability problems were defined if less than 70% of the participants were able to complete the tasks successfully [15,16]. The number of errors was recorded and counted. Errors were defined as unintended actions or mistakes made while attempting tasks [17]. These included, for example, the number of times the participants used the “back” button on their browsers or modified searches such as by removing keywords or filters.

The overall relative efficiency was assessed by the length of time (seconds) taken by the participants who successfully completed tasks and compared with the total time taken by all the participants [16,17]. A baseline target time was not established, as we expect users with a range of characteristics, such as familiarity and web-based comfort levels, to access this resource.

Satisfaction is a subjective measure of user attitudes and comfort while using a system. An overall usability problem was identified when the average SUS score was <68 or lower than the 50th percentile range [10]. To help provide a meaningful interpretation of individual and overall SUS ratings, an adjective rating scale was also used [18,19]. The interpretations of the SUS ratings are provided in the subsequent section and Multimedia Appendix 2. Semistructured interview questions were used to enhance the understanding of the participants’ SUS ratings.

The severity of website usability issues and errors was recorded as users thought aloud. Severity was defined as the impact of a problem during website navigation that prevented users from completing tasks successfully or efficiently [16]. Three levels of severity were reported: minor issues that caused annoyance, but the task could still be completed; serious issues that caused frustrations and may have caused users to abandon tasks; and critical issues that, when not fixed, impeded the ability to complete tasks.

Usability testing sessions and interviews were transcribed verbatim. Using an inductive approach, audio-visual recordings from the testing sessions and facilitator notes were reviewed to extract feedback on usability and experience. NVivo 12 Pro (QSR International) was used for coding the data. Test facilitators (AMLM and TJ) read and independently coded transcripts using an iterative open coding process. The results were compared to discuss codes and patterns, and the differences were resolved by discussion.

Think-aloud and semistructured interview data were analyzed through thematic analysis [20]. Thematic analysis is a useful and flexible approach for identifying, analyzing, and reporting patterns of meaning in qualitative data. The analysis began with the review of raw transcripts, facilitator notes, and semistructured interviews. Data were inductively and iteratively analyzed and coded using open coding to *chunk* data and apply descriptors, and axial coding was used to categorize codes and develop themes from categories [21]. A preliminary list of initial codes was developed and sorted into themes and supporting subthemes. To help form patterns and further conceptualize the subject, a constant comparison of the data was performed throughout the data analysis [22]. Themes were discussed with all the members of the research team and iteratively refined. Quotations and dialogues were extracted from the participant transcripts to illustrate core themes.

## Results

### Overview

A total of 10 participants were recruited into the study, of which 9 (90%) participants completed both the user testing session and semistructured interview, and 1 (10%) participant was unable to complete the testing session because of technological issues but was able to provide feedback about the website through an interview. One of the users completed the user testing session by telephone after attempting and noting technical difficulties with a tablet computer, and the other users participated through a laptop or desktop computer.

Overall, 2 cycles of usability tests were conducted by 2 facilitators to capture major challenges with usability. The first cycle of user tests was conducted with 7 participants, and the second cycle was conducted with 2 participants.

### Participant Characteristics

The participant demographics are presented in Table 1 (N=10). The sample consisted of 40% (4/10) of male participants and 60% (6/10) of female participants. Participants’ age ranged from 25 to 74 years, with 50% (5/10) aged 35 to 44 years, representing the largest age group. A total of 6 participants were identified as consumers, 1 as a caregiver, and 3 as health care providers. The participants reported that they experienced either one or a combination of visual, hearing, touch, physical (eg, mobility limitation and fatigue), and cognitive challenges. A total of 9 participants reported that they used a computer at home, and all the participants reported feeling very comfortable with using the internet. The participants reported preferring to seek health and service information from health care professionals (8/10, 80%), the internet (6/10, 60%), support agencies (4/10, 40%), and other sources (1/10, 10%); 30% (3/10) of participants reported having used websites or databases related to AT in the previous 3 months, such as an assistive device funding program within a province.

**Table 1:** Demographic characteristics of the study participants (N=10).

Characteristics		Values, n (%)
**Sex**		
	Male	4 (40)
	Female	6 (60)
	Other (please specify)	0 (0)
	Prefer not to say	0 (0)
**Age (years)**		
	18-24	0 (0)
	25-34	2 (20)
	35-44	5 (50)
	45-54	1 (10)
	55-64	0 (0)
	65-74	2 (20)
	>74	0 (0)
**User type**		
	Consumer	6 (60)
	Caregiver	1 (10)
	Health care provider	3 (30)
**Type of disability**		
	Visual	2 (20)
	Hearing	2 (20)
	Touch	1 (10)
	Physical (eg, mobility limitation and fatigue)	2 (20)
	Cognitive	1 (10)
	Mental health	0 (0)
	None reported	2 (20)
**Use of a computer at home**		
	Yes	9 (90)
	No	1 (10)
**Comfort level with using the internet**		
	Not at all comfortable	0 (0)
	A little comfortable	0 (0)
	Comfortable	0 (0)
	Very comfortable	10 (100)
**Preferred methods for seeking health or service information**		
	Health care professional	8 (80)
	Internet	6 (60)
	Support agencies (eg, governmental and nonprofit)	4 (40)
	Other (please specify)	1 (10)
**Used web-based health resources related to AT^a^<3 months**		
	Yes	3 (30)
	No	7 (70)

^a^AT: assistive technology.

### Usability Evaluation Findings

#### Task Performance Measures of Effectiveness

We measured task performance based on the ease of navigating the site for the participants unfamiliar with the website and the number of errors made (Multimedia Appendix 4). Participants attempted 164 tasks across all scenarios, with 120 tasks being successfully completed. A total of 175 errors were recorded. Errors included the number of times the participants restarted their search, removed keywords or filters after a search returned 0 results, and used the “back” button to return to the previous webpage.

In summary, 9 tasks were completed easily by the participants and had low error rates. These were tasks that asked participants to find information on government AT programs, government-funded programs, and charitable funding programs. The participants had the highest number of errors when performing the first task within each set of scenarios and when identifying charity programs within provinces. In general, some features were not initially obvious to the participants. For example, a participant was not aware that they could conduct a keyword search on the “Home” page and suggested including text to indicate this feature. Some participants reported that because this feature did not operate as expected (eg, did not return any matching programs when using keyword searching as they would using an internet search engine), they did not feel confident using the keyword search feature and that their frustration would likely result in them abandoning the use of the database and returning to methods that they were already familiar with; for example, using other search engines.

#### Task Performance Measures of Efficiency

Efficiency scores ranged from 29% (2/7) of participants successfully completing tasks with ease, to 100% (8/8) of participants completing tasks with ease. Of all tasks successfully attempted, only 4 tasks had an efficiency score of 100%. The efficiency scores tended to improve as the users continued to engage with the interface. As reflected by the overall scores and comments provided, the facilitators observed that the first few tasks across each scenario took participants longer to successfully complete than other tasks. Across scenarios, the participants were less efficient at finding charitable AT programs. For example, only 29% (2/7) of participants were successful in finding a charity program related to hearing impairment services in the Yukon Territory, without assistance. In another example, when asked to find a charity program in Ontario, only 63% (5/8) of participants were able to complete this task without assistance. Searching presented a challenge for the participants because they did not know what search terms to use or which filters to use or were confused by the language used on the website. Facilitators observed that the keyword search functions on the “Home” and main “Programs” pages were a source of frustration for many participants, who commented that this feature appeared to be less integrated and inconsistent in returning results.

#### Task Performance Measures of Perceived Satisfaction

The SUS scores from both the testing cycles are presented in Table 2. Scores >68 indicate average usability. The overall SUS score for this study was 62.22 or “OK/fair”, as described by Bangor et al [18,19]. Most participants provided an SUS rating described as fair or higher. The first cycle received an average SUS score of 59.3 (SD 17.48; OK or fair), with 29% (2/7) of participants providing a rating described as poor or lower. After cycle 1, revisions addressing usability issues were made to the website, specifically addressing areas related to presentation, functionality, and language. Furthermore, 22% (2/9) of additional participants were involved in user testing after these updates and provided an average SUS score of 72.5 (SD 3.54; good), indicating an improvement in comfort with the website.

During the 2 cycles of user testing, the severity of website usability issues and errors were reported, and feedback was summarized into 3 areas: presentation (eg, visual difficulties or issues when navigating the website), functionality (eg, issues impacting the ability to use or navigate the website), and language (eg, messages or meanings that users had a difficult time understanding or interpreting). Textbox 1 provides a summary of the major modifications made because of the user observations and feedback.

Several users provided suggestions for refining the design elements of the website to enhance intuitiveness and accessibility, which were adopted into subsequent iterations. This feedback was related to textual spacing, font size, and color contrast in accordance with the Canadian National Institute for the Blind and WCAG 2.0 accessibility guidelines, placement of the search filters, and refinement of the list of displayed results. A participant described that they sometimes felt overwhelmed by the amount of information presented on the website and returned searches; for example, the formatting of the “Programs” page and the result list was commented on the most by several users. Specifically, users provided suggestions for where to place prompts to reduce scrolling, such as “displaying number of matching results,” whereas other participants suggested additional text to help instruct users to locate returned results. Other suggestions to improve the result list included adding additional cues, such as indents and borders between results to indicate the separation of results, particularly as the length of some program names, while detailed, could present issues with readability, and improving the color contrast on the filter function to indicate when filters and subfilters have been selected. A user stated the following about overlapping text and illustrations:

It is not user friendly...because it forces you to distinguish, to make effort in distinguishing between various layers. And if you have vision loss, that is an additional effort that you shouldn’t have to make.

Most challenges with the functionality of the website were considered minor issues largely related to (1) the use of the filters (eg, leading to increased scrolling in the menu on the “Programs” page), (2) the inability to select a filter if a specific area was not selected, and (3) the unreliable functionality of the “clear all” feature. Feedback on some of the language or terminology used on the website was also provided, such as recommending using less technical language. For example, one of the participants said the following:

I question if everyone would understand what the “program,” “assistive technology” mean. [M]aybe it would be better to have a description on that.

**Table 2:** System usability scale (SUS) scores and corresponding grades.

SUS scores			Percentile range		Adjective
**Cycle 1: individual scores**					
	87.5	96-100		Best imaginable	
	50	N/A^a^		Poor	
	62.5	15-34		OK or fair	
	65	41-59		OK or fair	
	55	N/A		OK or fair	
	30	N/A		Worst imaginable	
	65	41-59		OK or fair	
**Cycle 1 average score, mean (SD)**					
	59.3 (17.48)	15-34		OK or fair	
**Cycle 2: individual scores**					
	75	70-79		Good	
	70	41-59		Good	
**Cycle 2 average score, mean (SD)**					
	72.5 (3.54)	60-64		Good	
**Overall score (cycles 1 and 2), mean (SD)**					
	62.2 (16.27)	15-34		OK or fair	

^a^N/A: not applicable.

**Summary of major modifications made to AccessATCanada.**

**Presentation**

- Included instructions on how to conduct a search on the “Home” page
- Improved font contrast and size across the website
- Minimized the instances where text overlapped graphics to improve readability
- Included additional labels and content description for the filters on the “Programs” page
- Made the selected filters more apparent by improving highlighting
- Improved readability by increasing spacing and adding dividers between search results
- Improved the presentation of the search results to reduce scrolling
- Made the search button more intuitive by repositioning it under the search bar rather than at the end of the filters list
- Displayed the total number of matching results at the top of the search results list

**Functionality**

- Addressed the issues with the *clear all* and *search* functionalities on the “Programs” page
- Made the bars of the filters one selection area to enable users to click anywhere to make their selection
- Improved the query process to fix the issue of high filter sensitivity

**Language**

- Website reviewed for the use of lay language
- Continual efforts made to review the resource pages and make information less ambiguous

### Themes

The key usability findings from the think-aloud and semistructured interviews were organized into the following themes: functionality and added value, the discovery of new programs and resources, and design aesthetics.

#### Functionality and Added Value

Overall, the participants commented that the website had features that were positive, “straightforward*,*” “intuitive,” and “easy to learn.” The filters were stated to be one of the most useful features on the website with respect to clear filter choices and navigation, clear understanding of drop-down menus, the population of the selected filters and search criteria in a single area, and the ability to clear filters or search criteria easily. For example, a user commented that the use of filters made searching more effective:

The thing I liked about it best though was [...] just being able to say, ok lets take these [filters] out [...] and see what it looks like. That was really well done. I don't think I’ve seen that before, so either I'm really ignorant or you’ve got a really neat tool to use.

Some participants also found the interactive map of Canada on the “Home” page to be an interesting feature. Several participants provided positive feedback about the icons indicating the type of AT available from the program on their information pages, stating that they were helpful.

Several participants provided suggestions that might further the beneficial impact of the website. For example, a participant observed that local programs within their province were not included in the database and suggested that smaller, “municipally based” programs may be more beneficial for clients to be connected to, as they “are more likely to get funding, because they are so local.” Moreover, 2 participants suggested expanding the eligible population groups (eg, youth students), and adding filters that allow users to find community-based health centers and assessment locations and programs that provide equipment rentals. One of the participants explained that one of the biggest barriers to accessing ATs was the financial barrier and finding information related to funding and suggested filling this gap by including funding applications in a simplified way.

#### Discovery of New Programs and Resources

Many participants described the website as a valuable resource for centralizing information about supports and programs related to AT and discovering new programs. Across the consumer, caregiver, and health care provider groups, the participants largely described the website as a “one-stop shop” that provides access to accurate information. For example, a participant stated the following:

I think the best feature is that it has all the information you need that’s all funding service programs, instead of going to multiple websites to find information, it has it down in one spot and you can filter for what you need to.

The participants stated that this website would be useful for individuals, particularly consumers and health care providers, as an additional tool to search for funding opportunities across different provinces and refer clients to. A participant, who was very familiar with their provincial resources because of their specific area of practice in AT, stated that the website would be helpful for new health care professionals and providers in general areas of practice for finding new resources and improving familiarity with the available programs:

I think if I was more of a generalist, like a community [occupational therapist] or maybe if I was working in a hospital and kind of supporting people and connecting to people to resources before going back into the community, I would probably use it.

#### Design Aesthetics

Most challenges with presentation, such as those related to website layout and general appearance, were reported as “minor issues” that caused annoyance but were not severe enough to hinder users from completing their tasks. The users identified areas to improve the formatting of the content for the ease of navigation and readability and ways to minimize the number of actions required to obtain a search result and improve readability by altering the color and contrast of the on-screen text. For example, the participants commented on the intuitiveness of where URL program links and the number of returned results were placed, spacing between the text of search results, amount of information presented, and the need to better differentiate between the chosen filters.

## Discussion

### Principal Findings

This study describes the methods and results of the usability testing of AccessATCanada, a web-based resource for improving access to information about AT programs and services in Canada. The usability of AccessATCanada was evaluated to understand how participants of different abilities would interact with the website and to identify and address major usability problems through navigating case scenarios. Although the overall SUS score was lower than the 68th percentile benchmark, the scores are subjective to the participants’ prior history of navigating AT programs, knowledge of services, and previous resources that they have used. In addition, it is important to note that because of the small sample size, these results are likely skewed. For example, the first participant in the study gave a high SUS score, which was noted as an outlier. The participants may have also given a lower score because of challenges experienced during usability testing, such as inconsistencies in the filter results. The overall evaluation of the beta version of the web-based resource tested over 2 cycles revealed positive experiences, such as the ease of navigation, clean layout, and value, which validated the objective of AccessATCanada, and opportunities to incorporate feedback to improve user experience and usability, such as searching, terminology, and accessibility challenges.

The current state of equitable access to available resources and funding opportunities in Canada and the complexity of navigating the AT system were catalysts for the creation of this resource. Our project fits into the growing response by providing a central, easy-to-use resource for various regional and national programs. This resource has the potential to identify and highlight areas where gaps exist, which might further reduce inequitable access to AT, specifically with respect to eligibility age, the types of AT and type of AT programs available, sources of funding (eg, charitable programs, government insurance programs, and other resource programs), conditions, and target population groups (eg, programs specific to Indigenous peoples, Veterans, refugees, and people who are incarcerated) within each province and territory. The ability to clearly identify the needs in these areas will be useful for priority planning for government programs and leaders working in this space.

An important area of consideration pertains to database maintenance and updating resource pages with the most recent and relevant information, particularly with resources that capture programs that are subject to changes in government regulations, such as AT. Two participants expressed concern about the long-term maintenance and sustainability of the database. For example, one of them described frustration with previous experiences with national databases running out of funding and failing to update their information. The other participant stated that keeping the website updated would encourage clinicians and end users to continue using the website. In recognition of this, the quality improvement of AccessATCanada has been planned as an ongoing process to ensure that the website is continuously updated and improved to provide up-to-date and accurate information.

It is also worth noting that outside the Canadian context, other countries, including those in less resourced settings, have also begun to respond to the need to improve access to AT by developing similar mobile apps and web-based resources [23,24]. Similar country-level or regional information search engines have been developed to provide publicly available information for end users and providers, such as Europe’s Global Assistive Technology Information Network, Denmark’s Assistive Technology Data-Denmark, Australia’s National Equipment Database, and South Africa’s Assistive Technology Database, which provides AT-related information for 10 other African countries.

Considering the aim of this website and that similar resources are to be usable by a broad range of people with and without different types of disabilities, user testing revealed the importance of including the ultimate end users during website development. As consumers and health information increasingly move to the web, previous studies have also suggested that usability testing is an important consideration for designers and developers [25,26]. Similar to other studies, we found that user experiences were reported more positively when website presentation and layout were considered and met WCAG 2.0 standards, such as the amount of text on a page, contrast, and reducing overlap between text and graphics [25,27,28]. The COVID-19 pandemic has illustrated just how critical web-based and digital experience has become and has especially highlighted the need to consider accessibility as an ongoing effort. For example, although AccessATCanada was built according to the WCAG 2.0 guidelines, the participants were still able to identify areas where meeting these guidelines could be improved.

We were unable to include users with significant technological challenges that might have been resolved if testing was conducted in person by providing appropriate technical support or guidance. This included engaging users with different disabilities in usability testing, for example, users who used eye-tracking technology, such as Tobii devices. This highlights an area where usability testing approaches can be improved to make products more user centered and inclusive for people of all abilities. For example, Asan and Yang [29] noted that there were few real-world usability evaluations that included eye-tracking technologies. Researchers, particularly those who conduct research in the areas of AT and disability supports, can more thoughtfully consider approaches for collecting data in time-sensitive environments from participants with various abilities.

Outside this resource, a lesson learned relates to centering website features on user needs, literacy, and common language. For example, although the participants expressed that the filter option was one of the best features, our early iteration revealed that when participants applied filters or used keywords that felt intuitive to them, the number of results returned was severely limited. During testing, the participants noted that keyword searching was not inclusive of different word variations, for example “wheelchair” and “wheel chair.” Likewise, there remains an issue regarding terminology that creates challenges in finding AT funding, as identified by participants and highlighted in previous studies [30]. The participants identified that some of the terminology used within the website was unclear, for example, “jurisdictions” or “government-legislated insurance programs,” and although we addressed this to the best of our ability, some language used was maintained to provide information continuity. The users emphasized the importance of using a common lay language that is easy for people to understand. However, inconsistent language may then present an accessibility challenge and could lead to avoidance of participating and applying for the funding service they may need. Language consistency is also important in current and future policy and program creations, as it could ultimately impact who can understand and access the available AT services within Canada. Although these issues were fixed in later iterations, these observations may benefit those interested in developing similar resources before launch.

### Limitations

Limitations were considered within the context of the study. First, recruitment was conducted during the early phases of the COVID-19 pandemic. A key recruitment strategy involved engaging project partners to share recruitment material with those who used their services; however, these partners were understandably prioritizing the urgent needs of their clients during this time. Although the authors had the intention of recruiting a larger number of individuals for the study, another cycle of usability testing could not be conducted, which may have impacted the evaluation of the website. However, despite its small sample size, in combination with the “think-aloud” technique, this study was able to identify major areas of improvement that were valuable in improving and directing updates for the website regarding its usability and functionality [31].

Second, because of the remote nature of the study, some participants experienced technological challenges that were difficult for researchers to address remotely, which may have impacted their engagement in usability testing. The participants may not have been able to complete the scenario tasks because of these difficulties, as technology may have contributed to potential feelings of frustration. In addition, all the participants in the study reported that they were “very comfortable” with internet use, which may have impacted their experience of navigating the website compared with those who may have lower comfort. Other considerations were that the participants were able to self-select browsers and devices to use while participating in the usability testing study, which could have led to differences in the website layout. Website usability was also not explicitly tested with participants who used AT devices to navigate the internet, such as eye-tracking or text-to-speech technology.

Considering these limitations, future research may recruit a greater variety of stakeholders and users with different abilities and levels of knowledge about AT. Further iterations of the website based on participant feedback will positively enhance the website’s usability and functionality.

### Conclusions

A critical lack of information about available types, programs, and funding opportunities is a significant barrier to accessing AT. AccessATCanada was developed as a first attempt at creating a resource to map and centralize information on AT programs and funding organizations in Canada. This study used an iterative approach to the usability testing of an innovative digital resource involving people with different disabilities to evaluate its effectiveness, efficiency, and end-user satisfaction and experiences. Usability testing is useful for incorporating user perspectives in the design process, assessing satisfaction, and identifying areas for iterative refinement of technology among a wider range of users [27]. This study highlights the value and elements that are key to the creation of user-centered resources. The goal of creating an easily searchable and functional website was supported by the results of usability testing metrics and feedback, which were used to develop and enhance the website. Although information provision and enhancing awareness about the types of available AT programs are essential steps to improve access to AT, equitable access remains a key policy issue in Canada and abroad, and further efforts are required to meet the needs of end users and caregivers who rely on AT the most.

## Appendix

Multimedia Appendix 1 Scenarios and tasks.

Multimedia Appendix 2 System Usability Scale interpretation tables.

Multimedia Appendix 3 Semistructured qualitative interview questions.

Multimedia Appendix 4 Task performance effectiveness findings.

## Abbreviations

AT: assistive technology
SUS: System Usability Scale
WCAG: Web Content Accessibility Guidelines

## References

[1] Schreiber D, Wang RH, Durocher E, Wilson MG (2017). Access to assistive technology in Canada: a jurisdictional scan of programs. University of Toronto.

[2] (2018). World health assembly resolution 71.8 improving access to assistive technology. World Health Assembly.

[3] World Health Organization, United Nations Children’s Fund (2022). Global Report on Assistive Technology. World Health Organization.

[4] Wang R, Wilson M, Putnam M, Bigby C (2021). Access to assistive technology in Canada. Handbook on Ageing with Disability.

[5] Mattison CA, Wilson MG, Wang RH, Waddell K (2020). Enhancing equitable access to assistive technologies in Canada: insights from citizens and stakeholders. Can J Aging.

[6] Penton V, Gustafson DL (2014). Access to assistive technology and single entry point programs. Can J Disabil Stud.

[7] Wang RH, Zdaniuk N, Durocher E, Wilson MG (2022). Policymaker and stakeholder perspectives on access to assistive technologies in Canada: challenges and proposed solutions for enhancing equitable access. Disabil Rehabil Assist Technol.

[8] (2019). Assistive Technology Funding and Service Programs in Canada. AccessATCanada.

[9] (2018). Ergonomics of human-system interaction — Part 11: Usability: Definitions and concepts - ISO 9241-11:2018. International Organization for Standardization.

[10] Brooke J, Jordan PW, Thomas B, McClelland IL, Weerdmeester B (1996). SUS: a 'quick and dirty' usability scale. Usability Evaluation in Industry.

[11] Rubin J, Chisnell D, Rubin J, Chisnell D, Spool J (2008). Chapter 9: Conduct the test sessions. Handbook of Usability Testing: How to Plan, Design, and Conduct Effective Tests. 2nd edition.

[12] Keenan SL, Hartson HR, Kafura DG, Schulman RS (1999). The usability problem taxonomy: a framework for classification and analysis. Empir Softw Eng.

[13] Khajouei R, Peute LW, Hasman A, Jaspers MW (2011). Classification and prioritization of usability problems using an augmented classification scheme. J Biomed Inform.

[14] Sousa VE, Dunn Lopez KD (2017). Towards usable e-health. A systematic review of usability questionnaires. Appl Clin Inform.

[15] Neilsen J, Budiu R (2001). Success rate: the simplest usability metric. Nielsen Norman Group.

[16] Rubin J, Chisnell D, Rubin J, Chisnell D, Spool J (2008). Chapter 11: Analyze data and observations. Handbook of Usability Testing: How to Plan, Design, and Conduct Effective Tests. 2nd edition.

[17] Mifsud J (2015). Usability Metrics - A Guide To Quantify The Usability Of Any System. Usability Geek.

[18] Bangor A, Kortum P, Miller J (2009). Determining what individual SUS scores mean: adding an adjective rating scale. J Usability Stud.

[19] Sauro J (2011). A Practical Guide to the System Usability Scale: Background, Benchmarks & Best Practices.

[20] Fereday J, Muir-Cochrane E (2006). Demonstrating rigor using thematic analysis: a hybrid approach of inductive and deductive coding and theme development. Int J Qual Methods.

[21] Bradshaw C, Atkinson S, Doody O (2017). Employing a qualitative description approach in health care research. Glob Qual Nurs Res.

[22] Boeije H (2002). A purposeful approach to the constant comparative method in the analysis of qualitative interviews. Qual Quant.

[23] Visagie S, Matter R, Kayange G, Chiwaula M, Harniss M, Kahonde C (2019). Perspectives on a mobile application that maps assistive technology resources in Africa. Afr J Disabil.

[24] Karki J, Rushton S, Bhattarai S, De Witte L (2021). Access to assistive technology for persons with disabilities: a critical review from Nepal, India and Bangladesh. Disabil Rehabil Assist Technol (forthcoming).

[25] Foley A (2011). Exploring the design, development and use of websites through accessibility and usability studies. J Educ Multimed Hypermedia.

[26] Goldberg L, Lide B, Lowry S, Massett HA, O'Connell T, Preece J, Quesenbery W, Shneiderman B (2011). Usability and accessibility in consumer health informatics current trends and future challenges. Am J Prev Med.

[27] Barbara AM, Dobbins M, Haynes RB, Iorio A, Lavis JN, Raina P, Levinson AJ (2016). The McMaster optimal aging portal: usability evaluation of a unique evidence-based health information website. JMIR Hum Factors.

[28] (WCAG) Overview. Web Content Accessibility Guidelines.

[29] Asan O, Yang Y (2015). Using eye trackers for usability evaluation of health information technology: a systematic literature review. JMIR Hum Factors.

[30] Jarvis T, Wang RH, Wilson MG (2020). Dialogue summary: implementing a policy vision for enhancing equitable access to assistive technologies in Canada. McMaster Health Forum, McMaster University.

[31] Nielsen J (2000). Why you only need to test with 5 users. Nielsen Norman Group.

