# Supplementary material for: Web-Based System Navigation Database to Support Equitable Access to Assistive Technology: Usability Testing Study
Source: JMIR Form Res. 2022 Nov 3;6(11):e36949. doi: 10.2196/36949 (PMC9673003; doi:10.2196/36949)
Supplement: Multimedia Appendix 1 [file formative_v6i11e36949_app1.docx]

**Multimedia Appendix 1. Scenarios and tasks**

**Scenario 1**

You live in Ontario. You are seeking funding and resources to get a new wheelchair as your needs have changed. You are no longer able to self-propel long distances and would like to explore getting a powered wheelchair. You are 59 years old and have been using a manual wheelchair since being diagnosed with Multiple Sclerosis 20 years ago. You work part time owing to the episodic nature of the condition. What government and charity programs might provide funding for your needs? What other sources of funding might you need to explore prior to seeking funding from government programs?

**Tasks**

- Find programs specific to powered wheelchairs
- Find sources of funding to explore related to powered wheelchairs in Ontario
- Find charity program(s) about wheelchairs in Ontario
- Navigate to resource about charity funding of wheelchairs in Ontario
- Find resource on government funding of wheelchairs in Ontario
- Navigate to government program(s) related to wheelchairs in Ontario

**Scenario 2**

You live in Nova Scotia and your 76-year-old mother is experiencing changes in her vision owing to macular degeneration. You are unfamiliar with the condition and would like to seek resources and information on funding for any technology or services that might assist in her day-to-day activities.

**Tasks**

- Find programs specific to vision devices
- Find sources of funding to explore related to vision devices in Nova Scotia
- Find charity program(s) about vision devices in Nova Scotia
- Navigate to resource about charity funding of vision devices in Nova Scotia
- Find resource on government funding about vision devices in Nova Scotia
- Navigate to government program(s) about vision devices in Nova Scotia

**Scenario 3**

You are a 68-year-old Alberta resident living with chronic physical and cognitive impairments after your stroke 15 years ago. Your spouse, whom you live with in a low-rise apartment, quit her part-time job to take care of you after the stroke. Your income has been very limited, including pensions and other resources, and is $35,000 per year. Because of changes in your health and increased inactivity you have gained weight, making mobility and care more difficult. You would like to find additional supports at home to enable your wife to more easily care for you and to explore mobility and home care equipment (e.g., for bathing, toileting, etc.) that can make things easier.

**Tasks**

- Find programs specific to home care in Alberta
- Find programs specific to mobility equipment and supports in Alberta
- Find resources regarding eligibility for home care in Alberta based on your income
- Find sources regarding eligibility for mobility equipment and supports in Alberta based on your income
- Find charity program(s) about mobility equipment and supports

**Scenario 4**

You are a health professional/service worker in the Yukon Territory. A client has inquired about the need for a hearing device due to reduced hearing. Assuming you know very little about hearing devices, where would you go to find information resources about organizations or financial help on that subject? What programs and services would you refer consumers to based on their needs?

**Tasks**

- Find programs located in the Yukon
- Find a charity program related to hearing impairment services
- Navigate to eligibility criteria regarding service delivery of hearing impairment services
- Navigate to funding eligibility criteria regarding hearing impairment services
