# Supplementary material for: Web-Based System Navigation Database to Support Equitable Access to Assistive Technology: Usability Testing Study
Source: JMIR Form Res. 2022 Nov 3;6(11):e36949. doi: 10.2196/36949 (PMC9673003; doi:10.2196/36949)
Supplement: Multimedia Appendix 2 [file formative_v6i11e36949_app2.docx]

**Multimedia Appendix 2**

Table 1. System usability scores (SUS) questionnaire items and grading scale [10]

| System Usability Scale | Strongly Disagree |  |  |  | Strongly Agree |
| --- | --- | --- | --- | --- | --- |
| Items | (1) | (2) | (3) | (4) | (5) |
| 1. I think that I would like to use this system frequently |  |  |  |  |  |
| 2. I found the system unnecessarily complex |  |  |  |  |  |
| 3. I thought the system was easy to use |  |  |  |  |  |
| 4. I think that I would need the support of a technical person to be able to use this system |  |  |  |  |  |
| 5. I found the various functions in this system were well integrated |  |  |  |  |  |
| 6. I thought there was too much inconsistency in this system |  |  |  |  |  |
| 7. I would imagine that most people would learn to use this system very quickly |  |  |  |  |  |
| 8. I found the system very cumbersome to use |  |  |  |  |  |
| 9. I felt very confident using the system |  |  |  |  |  |
| 10. I needed to learn a lot of things before I could get going with this system |  |  |  |  |  |

Table 2. System usability scores and corresponding grades [18,19]

| Grading SUS Key | Adjective |
| --- | --- |
| 92 | Best imaginable |
| 85 | Excellent |
| 72 | Good |
| 52 | OK/Fair |
| 38 | Poor |
| 25 | Worst imaginable |
|  |  |
| Percentile ranks | Grades |
| >81 | A |
| 68-81 | B |
| 68 | C |
| 51-67 | D |
| <51 | F |
