# Supplementary material for: Web-Based System Navigation Database to Support Equitable Access to Assistive Technology: Usability Testing Study
Source: JMIR Form Res. 2022 Nov 3;6(11):e36949. doi: 10.2196/36949 (PMC9673003; doi:10.2196/36949)
Supplement: Multimedia Appendix 3 [file formative_v6i11e36949_app3.docx]

**Multimedia Appendix 3. Semi-structured qualitative interview questions**

1. How would you describe your feelings when using the database?
2. You answered that you found that system unnecessarily complex. Could you please describe what you found complex?
3. Could you please elaborate on your rating of inconsistency?
4. What do you feel you need to learn before being able to use this system?
5. What did you least like about the system (what was the worst feature)?
6. What did you most like about the system (what was the best feature)?
