# Supplementary material for: Web-Based System Navigation Database to Support Equitable Access to Assistive Technology: Usability Testing Study
Source: JMIR Form Res. 2022 Nov 3;6(11):e36949. doi: 10.2196/36949 (PMC9673003; doi:10.2196/36949)
Supplement: Multimedia Appendix 4 [file formative_v6i11e36949_app4.docx]

**Multimedia Appendix 4.** Task performance effectiveness findings.

| Scenario and task number (N=189) | | Task description | Total number of tasks attempted (n=164) | Tasks not completed (n=44), n (%) | Tasks successfully completed (n=120), n (%) | Total errors (n=175) |
| --- | --- | --- | --- | --- | --- | --- |
| **1** | | | | | | |
|  | 1 | Find programs specific to powered wheelchairs | 9 | 4 (44) | 5 (56) | 33 |
|  | 2 | Find sources of funding to explore related to powered wheelchairs in Ontario | 9 | 3 (33) | 6 (67) | 4 |
|  | 3 | Find a charity program related to wheelchairs in Ontario | 8 | 3 (37) | 5 (63) | 12 |
|  | 4 | Navigate to resource about charity funding of wheelchairs in Ontario | 8 | 2 (25) | 6 (75) | 2 |
|  | 5 | Find resource on government funding of wheelchairs in Ontario | 8 | 0 (0) | 8 (100) | 4 |
|  | 6 | Navigate to government program(s) related to wheelchairs in Ontario | 8 | 0 (0) | 8 (100) | 9 |
| **2** | | | | | | |
|  | 1 | Find programs specific to vision devices | 9 | 4 (44) | 5 (56) | 20 |
|  | 2 | Find sources of funding to explore related to vision devices in Nova Scotia | 9 | 5 (56) | 4 (44) | 9 |
|  | 3 | Find a charity program related to vision devices in Nova Scotia | 9 | 4 (44) | 5 (56) | 6 |
|  | 4 | Navigate to resource about charity funding of vision devices in Nova Scotia | 6 | 2 (33) | 4 (67) | 5 |
|  | 5 | Find resource on government funding related to vision devices in Nova Scotia | 8 | 1 (12) | 7 (88) | 2 |
|  | 6 | Navigate to government program(s) about vision devices in Nova Scotia | 6 | 2 (33) | 4 (67) | 4 |
| **3** | | | | | | |
|  | 1 | Find programs specific to home care in Alberta | 8 | 3 (38) | 5 (62) | 9 |
|  | 2 | Find programs specific to mobility equipment and supports in Alberta | 7 | 1 (14) | 6 (86) | 3 |
|  | 3 | Find resources regarding eligibility for home care in Alberta based on your income | 7 | 2 (29) | 5 (71) | 13 |
|  | 4 | Find sources regarding eligibility for mobility equipment and supports in Alberta based on your income | 6 | 0 (0) | 6 (100) | 6 |
|  | 5 | Find charity program(s) about mobility equipment and supports | 7 | 1 (14) | 6 (86) | 4 |
| **4** | | | | | | |
|  | 1 | Find programs related to hearing located in Yukon | 9 | 1 (11) | 8 (89) | 7 |
|  | 2 | Find a charity program related to hearing impairment services | 7 | 5 (71) | 2 (29) | 17 |
|  | 3 | Navigate to eligibility criteria regarding the delivery of hearing impairment services | 8 | 0 (0) | 8 (100) | 4 |
|  | 4 | Navigate to funding eligibility criteria regarding hearing impairment services | 8 | 1 (12) | 7 (88) | 2 |
